# The influence of neuronal electrical activity on the mammalian central clock metabolome

**DOI:** 10.1007/s11306-018-1423-z

**Published:** 2018-09-17

**Authors:** M. Renate Buijink, Michel van Weeghel, M. Can Gülersönmez, Amy C. Harms, Jos H. T. Rohling, Johanna H. Meijer, Thomas Hankemeier, Stephan Michel

**Affiliations:** 1 Department of Cellular and Chemical Biology, Leiden University Medical Center, Einthovenweg 20, 2333 ZC Leiden, The Netherlands; 2 Analytical BioSciences and Metabolomics, Division of Systems Biomedicine and Pharmacology, Leiden Academic Centre for Drug Research (LACDR), Leiden University, Einsteinweg 55, 2333 CC Leiden, The Netherlands

**Keywords:** Circadian clock metabolites, Suprachiasmatic nucleus, Neuronal activity, Small brain samples, ZIC-cHILIC-MS

## Abstract

**Introduction:**

Most organisms display circadian rhythms in physiology and behaviour. In mammals, these rhythms are orchestrated by the suprachiasmatic nucleus (SCN). Recently, several metabolites have emerged as important regulators of circadian timekeeping. Metabolomics approaches have aided in identifying some key metabolites in circadian processes in peripheral tissue, but methods to routinely measure metabolites in small brain areas are currently lacking.

**Objective:**

The aim of the study was to establish a reliable method for metabolite quantifications in the central circadian clock and relate them to different states of neuronal excitability.

**Methods:**

We developed a method to collect and process small brain tissue samples (0.2 mm^3^), suitable for liquid chromatography–mass spectrometry. Metabolites were analysed in the SCN and one of its main hypothalamic targets, the paraventricular nucleus (PVN). Tissue samples were taken at peak (midday) and trough (midnight) of the endogenous rhythm in SCN electrical activity. Additionally, neuronal activity was altered pharmacologically.

**Results:**

We found a minor effect of day/night fluctuations in electrical activity or silencing activity during the day. In contrast, increasing electrical activity during the night significantly upregulated many metabolites in SCN and PVN.

**Conclusion:**

Our method has shown to produce reliable and physiologically relevant metabolite data from small brain samples. Inducing electrical activity at night mimics the effect of a light pulses in the SCN, producing phase shifts of the circadian rhythm. The upregulation of metabolites could have a functional role in this process, since they are not solely products of physiological states, they are significant parts of cellular signalling pathways.

**Electronic supplementary material:**

The online version of this article (10.1007/s11306-018-1423-z) contains supplementary material, which is available to authorized users.

## Introduction

Almost all organisms express circadian rhythms in physiology and behaviour defined by a period of about 24 h. In mammals, these circadian rhythms are controlled by the suprachiasmatic nucleus (SCN), a small brain area located just above the optic chiasm (Moore and Eichler 1972; Stephan and Zucker 1972). The SCN is synchronized to the 24-h environmental cycle mainly by light. In the absence of external cues like light or temperature cycles, the neuronal network of the SCN sustains an autonomous circadian rhythm in electrical activity and neurotransmitter release (Gillette and Reppert 1987; Nishino et al. 1976). These signals from the SCN serve as a temporal reference for the rest of the body and orchestrate circadian rhythms throughout the brain and body, including sleep-wake cycles (Ibuka and Kawamura 1975; Moore 2007), food intake and energy metabolism (Coomans et al. 2013; Kalsbeek et al. 2011; Nagai et al. 1978). Disturbances in circadian rhythms have been found to be associated with many diseases, including metabolic syndrome (Rudic et al. 2004; Turek et al. 2005) and neurodegenerative diseases (Ju et al. 2013; Kondratova and Kondratov 2012; Musiek et al. 2015; Wulff et al. 2010).

Neurons in the SCN exhibit robust autonomous rhythmicity, which is controlled on several levels. There are known intracellular oscillators on the level of gene expression, cytosol and electrical activity. First, on the level of gene expression, the “molecular clock”—consisting of clock genes and proteins—constitutes a well-described core transcription-translation feedback loop (TTFL), which has a cycle time of about 24 h (Sangoram et al. 1998). Second, in addition to the TTFL there are several cytosolic oscillators like Ca^2+^, cAMP and redox state (Ikeda et al. 2003; O’Neill et al. 2008; Wang et al. 2012). The redox oscillator is well-preserved among species and is considered to be a link between metabolic state, and both the molecular clock and membrane excitability (Wang et al. 2012). Lastly, an essential part of the SCN clockwork is the electrical activity of SCN neurons. Electrical activity modifies the molecular clock and vice versa and this interaction between membrane and clock genes is essential for sustained rhythm generation (Allen et al. 2017; Colwell 2011).

Neurons in the SCN are electrically active during the day, and relatively silent during the night in both diurnal and nocturnal animals (Inouye and Kawamura 1979; Sato and Kawamura 1984). One of the main targets for the SCN activity is the paraventricular nucleus (PVN) of the hypothalamus, which receives both paracrine as well as GABAergic and glutamatergic input from the SCN, and controls feeding behaviour and plasma glucose level (Kalsbeek et al. 2004; Santoso et al. 2017; Tousson and Meissl 2004). While the rhythm in electrical activity of extra-SCN areas in the hypothalamus has been found to be reversed in phase, the only study that directly measured electrical activity in the PVN has found the electrical activity rhythm to be in phase with the SCN (Inouye and Kawamura 1979; Kubota et al. 1981; Tousson and Meissl 2004).

**Fig. 1:**
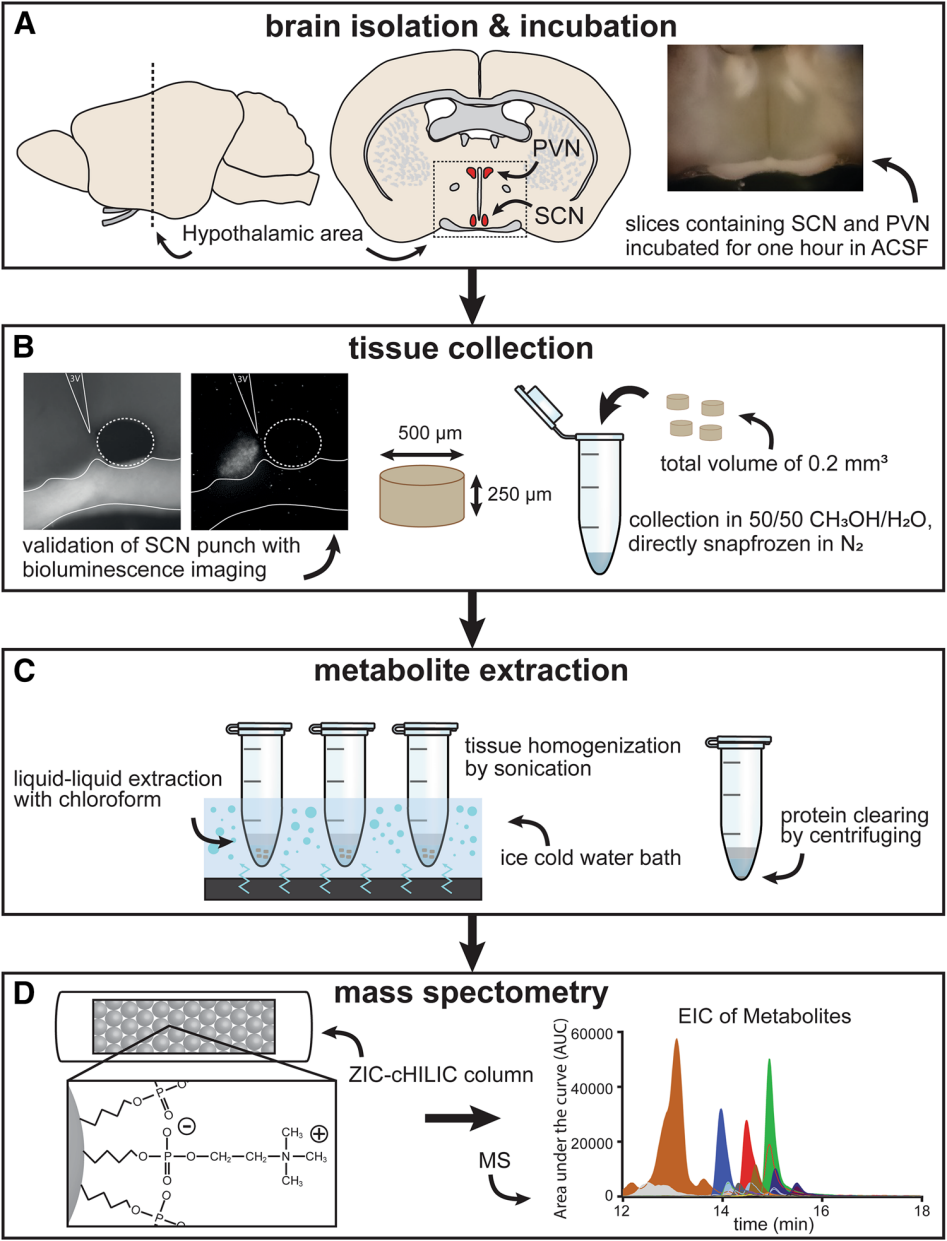
Overview of tissue sampling and processing. **a** For extraction and measurements of metabolites in SCN and PVN tissue, the brain was first isolated from the mouse, and the hypothalamic region, containing the SCN was cut in 250 µm thick slices. These slices were incubated in ACSF, or ACSF with either 0.5 µM TTX or 15 mM K^+^. **b** After incubation, the SCN and PVN were extracted from the slices by a sample corer. Because of the tight control over the thickness of the slices (250 µm) and the diameter of the punch (500 µm), the volume of the sample was constant (0.2 mm^3^). Punches were placed in 50/50 methanol/water and directly snap frozen in N_2_. Samples were kept at − 80 C until metabolite extraction. **c** Metabolites were extracted from the tissue by using a liquid–liquid extraction method with 100 µL chloroform added to the 100 µL 50/50 methanol/water. Samples were homogenized in an ice-cold sonication bath for 3 × 10 min. Between sonication, samples were snap frozen for a short period in N_2_. Proteins were cleared from the solution by centrifuging. The top layer was transferred to a clean 0.5 mL tube and dried in a vacuum concentrator. **d** The dried samples were then reconstituted in 20 µL of 60/40 methanol/water and analysed by liquid chromatography through a ZIC-cHILIC column, followed by mass spectrometry

Metabolites are products of cellular regulatory processes, and their relative levels are therefore indicative of the metabolic state of a cell. They are an attractive target to study since they are identical across species, and organisms differ less in their metabolome than in their genome or proteome. Several metabolites, such as cAMP (O’Neill et al. 2008), and metabolic pathways, such as the pentose phosphate pathway (Rey et al. 2016) are considered to be relevant for proper functioning of the circadian clockwork. Metabolomics has proven to be useful to study circadian clock function in liver and blood of mice (Eckel-Mahan et al. 2012; Minami et al. 2009), and has been applied to blood samples of humans to investigate the effect of sleep deprivation (Davies et al. 2014). However, until now there are no metabolomics studies of specific brain areas like the SCN, an important constraint being their small size, especially in commonly used animal models such as the mouse. Although there are complex techniques to measure metabolites with mass spectrometry in small samples, and even single cells (Lapainis et al. 2009; Qi et al. 2018), more generally applicable methods for small tissue samples are lacking. Therefore, we aimed to design a suitable method for tissue sampling and metabolite extraction for the analysis of metabolites with liquid chromatography–mass spectrometry (LC–MS) in small brain areas (Fig. 1). Using this method, we have studied the metabolic profiles of the SCN, as well as the PVN—a first relay area to control many physiological functions. We were interested in endogenous differences between day and night, as well as the effect of exogenously modulating neuronal activity on the metabolic profile of these brain areas.

Since we expected differences in energy metabolism, we focused our analytical approach on small polar metabolites, including metabolites from the TCA cycle, glycolysis, pentose phosphate and nucleotide pathways. For the metabolic profiling of the SCN and the PVN we used zwitterionic hydrophilic interaction liquid chromatography mass spectrometry (ZIC-cHILIC-MS), which is especially suitable for the analysis of anionic polar metabolites in complex aqueous matrices. In this study we show that it is feasible to measure metabolites in very small tissue samples (0.2 mm^3^). We could reliably measure and identify 35 metabolites in SCN and PVN tissue. We found only one metabolite (malate) significantly upregulated in the day compared to the night condition. Exposure to high extracellular K^+^ at midnight upregulated many of the metabolites measured in SCN and PVN tissue, including all metabolites of the TCA cycle.

## Method

### Animals and housing

This study was performed in accordance with the Dutch law on animal welfare. The permit was issued by the animal experiments committee Leiden (DEC 12250). Male C57BL/6 mice were held in the animal facility of the Leiden University Medical Center, with food and water available ad libitum. Mice were kept in 24-h light–dark cycles with 12 h of light (50–100 lx; Osram truelight TL) and 12 h of darkness. Mice were approximately 3 months old (84–91 days) at the time of the experiments.

### SCN and PVN tissue sampling

For the collection of SCN and PVN tissue, mice were sacrificed either an hour before midday (ZT6) or midnight (ZT18). The mice were fasted for 4 h before the start of the experiment. After decapitation, the brain was quickly dissected and placed in ice cold, low Ca^2+^ and high Mg^2+^ artificial cerebral spinal fluid (ACSF), containing (in mM): NaCl (116.4), KCl (5.4), NaH_2_PO_4_ (1.0), MgSO_4_ (0.8), CaCl_2_ (1.0), MgCl_2_ (4.0), NaHCO_3_ (23.8), d-glucose (15.1) and 5 mg/L gentamicin (Sigma Aldrich) saturated with 95% O_2_ − 5% CO_2_ (pH 7.4). From each individual animal, two consecutive coronal slices of 250 µm were cut with a VT 1000S vibratome (Leica). The brain slices were incubated for an hour in either normal ACSF containing 2 mM CaCl_2_ and no MgCl_2_ (ZT6; ZT18), or normal ACSF with TTX (0.5 µM; ZT6) or higher levels of K^+^ (15 mM; ZT18), at room temperature (*n* = 6 for all groups). This method, including the incubation in ACSF is widely used as preparation for physiological experiments on SCN tissue (Nakamura et al. 2012; Michel et al. 2013). Slices were kept cold while collecting the SCN and PVN punches within a few minutes after incubation. We collected bilateral punches of Ø 500 µm (sample corer, 19-gauge, Fine Science Tools; adapted from Lee et al. 2010), from two consecutive slices, resulting in 4 punches of both the SCN and PVN, with a total volume of 0.2 mm^3^ from one animal per sample. This standardized and reproducible volume rendered weighing the samples unnecessary. Punches were directly placed in 100 µL ice cold 50/50 methanol/H_2_O containing 5 µM of internal standards (succinic acid-D4, 13C5-valine, 13C4-15N2-asparagine, 13C5-glutamine and 15N2-UMP), and snapfrozen in liquid nitrogen (adapted from Nemes et al. 2011). The combination of methanol and snapfreezing ensured adequate quenching of enzymatic activity, is suitable for metabolite extraction, and can be used for the ZIC-cHILIC column. Samples were stored at − 80 °C until further processing. Figure 1b shows a bright field and a bioluminescence image from a brain slice with one of the two SCN cores removed by punch.

### Metabolite extraction

Metabolites were extracted using a combination of chemical and mechanical techniques (Fig. 1b, c). First, a liquid–liquid extraction (LLE) was performed by adding 100 µL of chloroform to the samples in methanol–water. Samples were vortexed, then sonicated in an ice-cold sonication bath for 10 min, followed by snapfreezing in liquid nitrogen. This procedure was repeated 3 times. The samples were then centrifuged for 10 min at 14.000 g at 4 °C to remove proteins from the solution. Before and after the sonication and centrifuging, samples were kept on ice. From the 100 µL polar top layer 80 µL was transferred to a 1.5 mL tube and dried in a vacuum concentrator (Labconco, MO, USA) for 3 h. The samples were reconstituted in 20 µL of methanol/H_2_O solution (60/40).

### Metabolite analysis

Metabolites were analysed using liquid chromatography followed by mass spectrometry. For the analysis, an Agilent 1200 ultra-high-pressure liquid chromatography system (Agilent technologies, Santa Clara, CA, USA) coupled to a SCIEX TripleTOF 5600 quadrupole-time-of-flight mass spectrometer (Framingham, MA, USA) was used. Samples were kept at 10 °C in the autosampler and 5 µL of sample was injected on the analytical column. The chromatographic separation was established using a SeQuant ZIC-cHILIC column (PEEK 100 × 2.1 mm, 3.0 µm particle size; Merck KGaA, Darmstadt, Germany), which was kept at 15 °C using a column thermostat (560-CIL, Cleuzeau Info Labo, France). The flow rate was 0.2 mL/min. The mobile phases where composed of (A) 90/10 acetonitrile/H_2_O with 5 mM ammonium acetate at pH 6.8 and (B) 10/90 acetonitrile/H_2_O with 5 mM ammonium acetate at pH 6.8, respectively. Metabolites were separated using a gradient composed of 100% A for 3 min; ramping 3–20 min to 36% A; ramping 28–28.5 to 100% A and re-equilibrated from 28.5 to 36 min with 100% A. The MS data was acquired at full scan range 50–800 amu at a scan rate of 10 scans/s in negative ionisation mode and the source temperature was kept at 400 °C.

### Data analysis and statistics

The metabolite peaks were integrated using MultiQuan Software v3.0 (SCIEX, Framingham, MA, USA). The responses of the targeted metabolites were corrected for the response of a selected internal standard. Statistical analysis was performed using GraphPad Prism 6 (Graphpad, La Jolla, CA, USA) and statistical programming language R (http://www.r-project.org), with packages ‘mixOmics’ (Rohart et al. 2017) for the PLS-DA, and ‘gplots’ (https://cran.r-project.org/package=gplots) to visualize the heatmaps. Differences in individual metabolites were evaluated using a one-way analysis of variance (ANOVA) with a Tukey post-hoc correction for multiple comparisons. A *P* value of < 0.05 was considered significant.

## Results

### ZIC-cHILIC-MS optimization for SCN and PVN

The ZIC-cHILIC-MS method was partly adapted from a previous application of the ZIC-cHILIC column for serum metabolite profiling of colorectal tumor patients (Zhu et al. 2014) and modified for the measurement of anionic polar metabolites in SCN and PVN samples. For the sensitive measurement of the anionic polar metabolites in these samples, we optimized the extraction procedure for small sample sizes as described in Sect. 2. After metabolite extraction from the SCN and PVN, we were able to semi-quantitatively determine 35 anionic polar metabolites in these samples (Table S1 and Fig. S1). Polar metabolites were retained on the ZIC-cHILIC column and chromatographic separation was obtained (Fig. S1). For most of the metabolites, there was base-line separation and sensitivity was high enough to perform semi-quantitative analysis on the measured metabolites when using appropriate reconstructed ion chromatograms (Table S1). To determine ion suppression, we performed a post-column infusion experiment and did not observe any significant ion suppression (data not shown).

### Endogenous and induced electrical activity affects metabolites in SCN and PVN tissue

To study the effect of time of day and of neuronal electrical activity in SCN and PVN tissue, we measured metabolites at two time-points, midday (ZT6) and midnight (ZT18), and in part of the brain slices we chemically manipulated the electrical activity. Intrinsic electrical activity in the SCN is high during the day, and low during the night, and the electrical activity in the PVN has been found to be in phase with the SCN (Tousson and Meissl 2004). Neuronal activity was blocked at midday with tetrodotoxin (TTX). TTX blocks Na^2+^-channels, preventing the initiation of action potentials, but does not affect membrane potential. Neuronal activity was induced at midnight by using increased extracellular K^+^ concentrations, which depolarizes the neuronal membrane, increasing the chance of the initiation of action potentials.

**Fig. 2:**
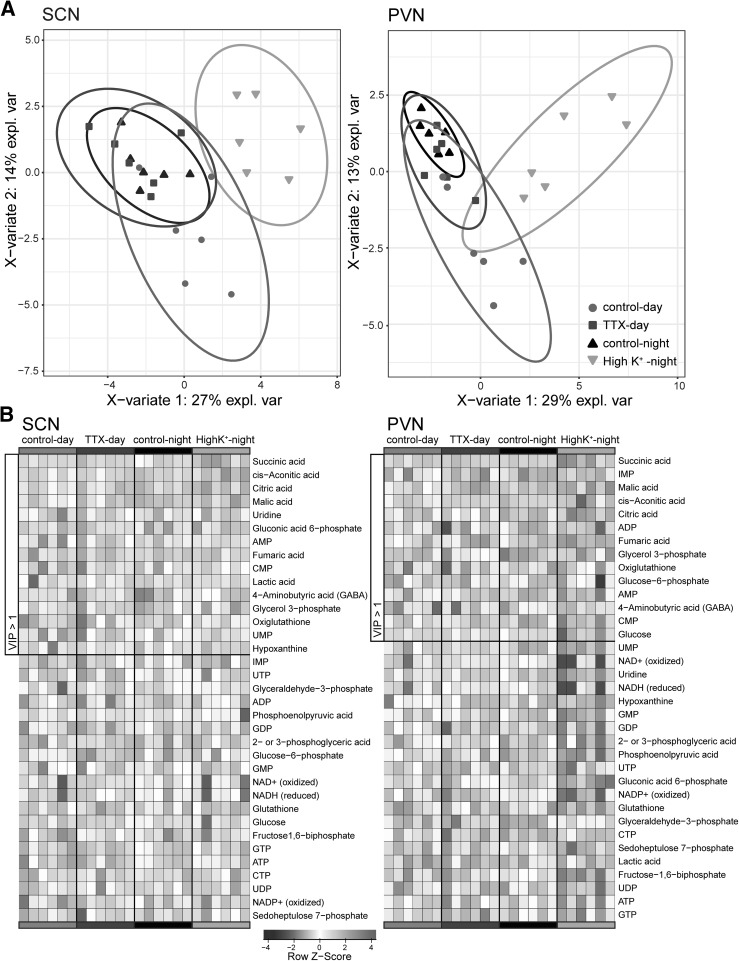
Global analysis of the complete data set. **a** An PLS-DA analysis separated the high K^+^-night group clearly, and to a lesser extend also separated the control-day group. The TTX-day and control-night groups largely overlap. Since silencing the neurons of the SCN with TTX at midday was intended to imitate the midnight state of the SCN this is in line with expectations. The results are similar for the SCN and PVN. **b** The contribution of the individual metabolites were calculated from the PLS-DA, giving a value of the VIP. The metabolites are ordered on their VIP score and their relative amount per individual sample shown in a heat plot. Among the metabolites with high VIP scores are all measurable metabolites of the TCA cycle, and several from the glycolysis pathway

In order to understand the effect of the changes in metabolite levels due to tissue treatment, we applied a PLS-DA analysis to the ZIC-cHILIC-MS dataset. This resulted in a clear separation of the night High K^+^ group and to a lesser degree of the control day group from the TTX and control night group (Fig. 2a). The TTX and control night group could not be separated by the PLS-DA, which is not unexpected, since the TTX manipulation was intended to simulate the night conditions for neurons in the SCN and PVN. The heatmap in Fig. 2b represents the data for all the identified and measurable metabolites for all biological samples of this study, ordered on Variable Importance in Projection (VIP) score for the PLS-DA (Chong and Jun 2005). Interestingly, all the intermediates of the TCA cycle were among the metabolites with the highest VIP score.

### TCA cycle intermediates are upregulated after exposure to high extracellular K^+^

**Fig. 3:**
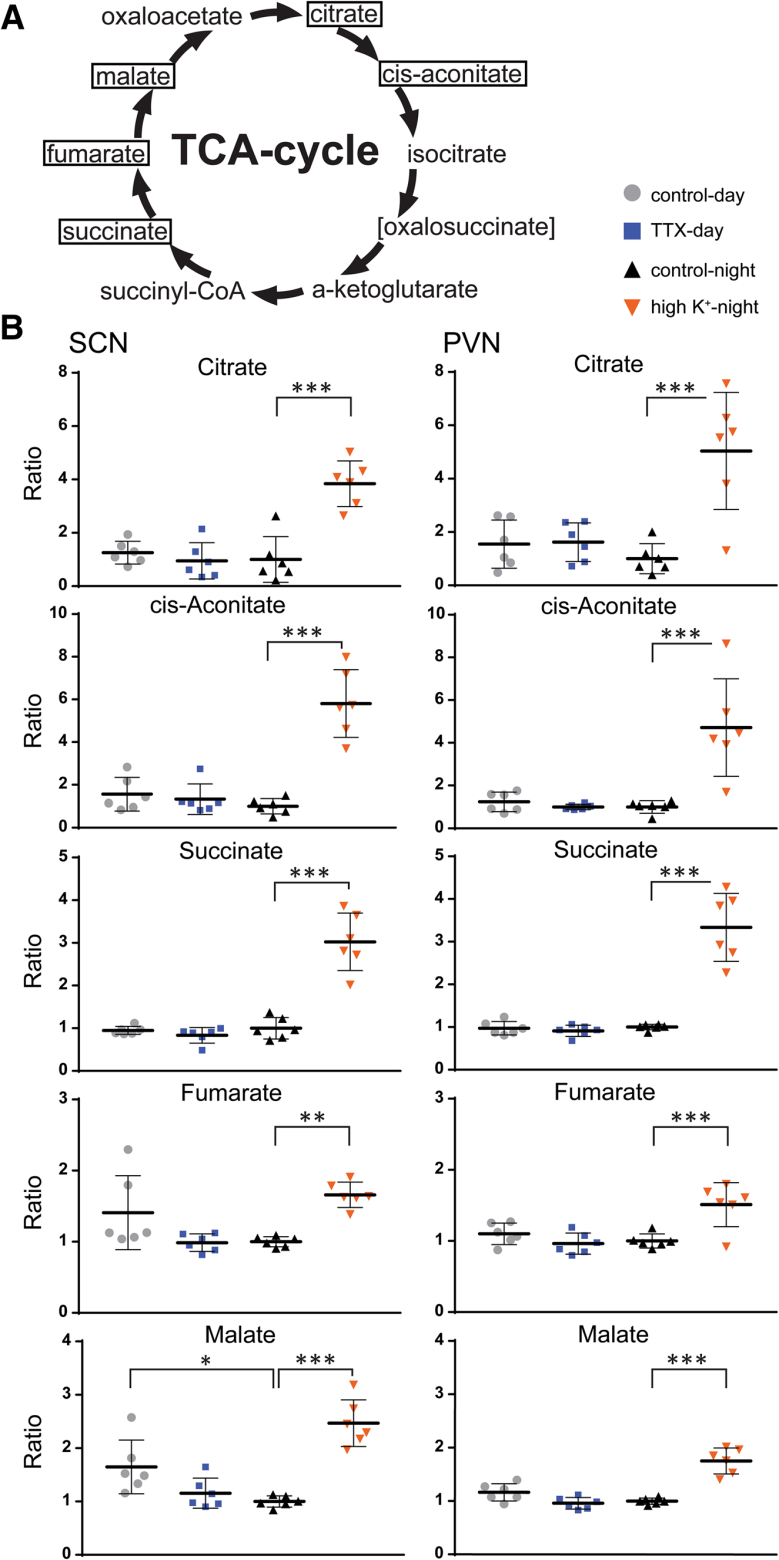
Metabolites of the TCA cycle are mainly affected by incubation in high K^+^. **a** From the main metabolites of the TCA cycle, five were reliably measurable with the ZIC-cHILIC-MS method (boxed). **b** In both the SCN and PVN, all these metabolites were significantly upregulated by incubation in high K^+^-medium at midnight. In the SCN, there was higher level of malate in the control day group, compared to control night. *P < 0.05, **P < 0.01, ***P < 0.001

Given the high VIP scores for the intermediates of the TCA cycle on the PLS-DA, we performed additional analysis on these metabolites (Fig. 3). From the metabolites directly involved in the TCA cycle, we could measure citrate, cis-aconitate, succinate, fumarate and malate, which were all upregulated by exposure to high extracellular K^+^, compared to the other groups, both in the SCN and PVN (citrate, cis-aconitate, succinate, malate *P* < 0.0001, fumarate *P* < 0.01). Malate was significantly higher in the control-day group, compared to the control-night group (*P* < 0.5). Thus, upregulating electrical activity by exposure to higher extracellular K^+^ levels resulted in a strong upregulation of all measurable intermediates of the TCA cycle. In samples taken at midday compared to midnight, we could detect a significant difference for the TCA intermediate malate only.

## Discussion

In this study we demonstrate the feasibility to measure metabolites in small tissue samples with ZIC-cHILIC-MS. We were able to semi-quantitatively measure 35 metabolites in the small tissue samples of the SCN and PVN (Table S1). Furthermore, we show that exposure of SCN and PVN tissue to high extracellular K^+^ levels at night results in upregulation of many of the measured metabolites, and in particular of TCA cycle intermediates (Fig. 3; Figs. S2–S5).

**Fig. 4:**
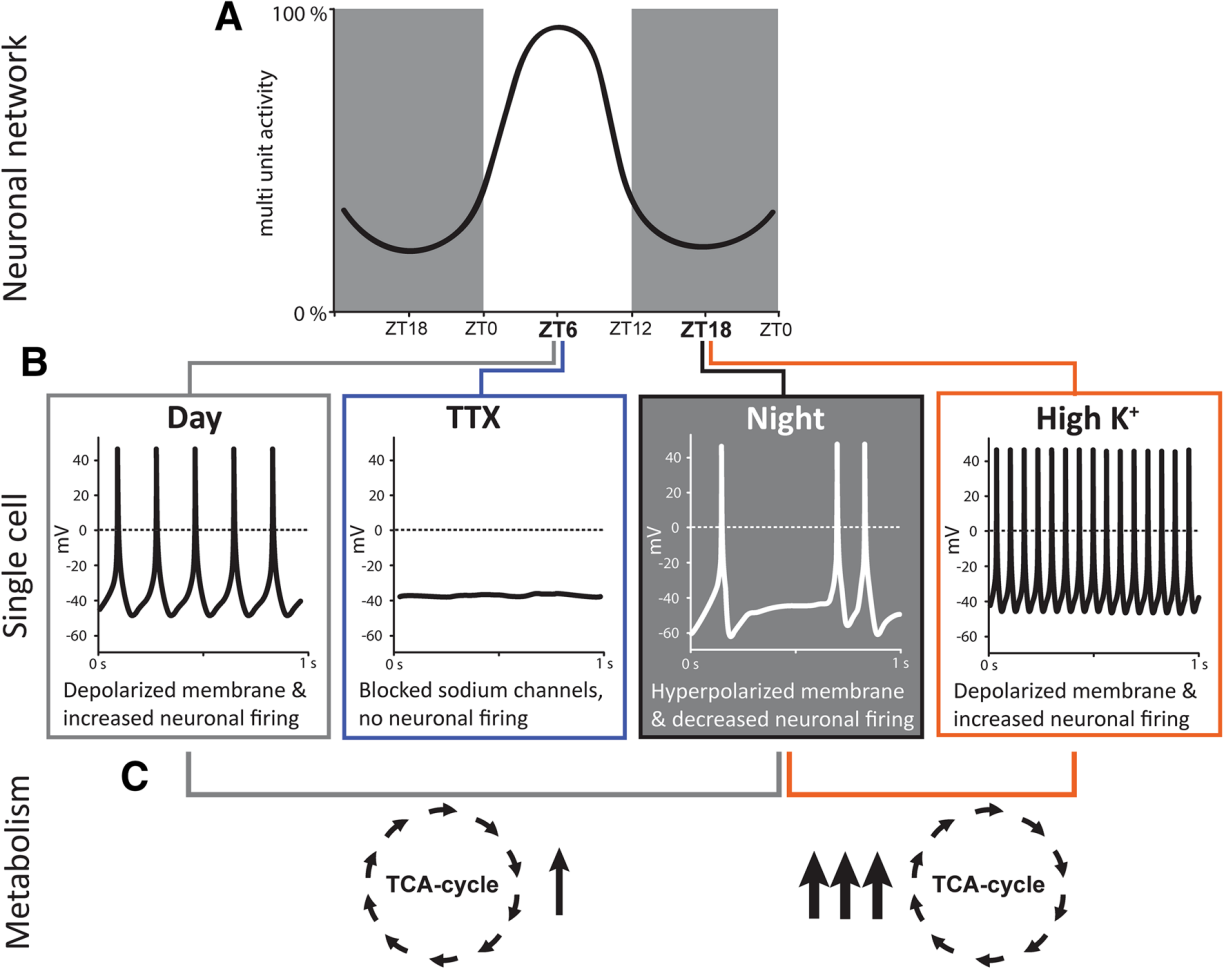
Schematic overview of electrical activity in the SCN at the time of the experiments and its effect on metabolism. **a** On the network-level, the multi-unit activity of the SCN reaches its peak during the light period, between ZT0 (start light period) and ZT12 (end light period). **b** At the time of sampling, at ZT6 (day), most individual neurons are electrically active, and fire at a frequency of around 8 Hz. At this time point, neurons were completely silenced by applying tetrodotoxin, which blocks sodium channels. At the second sampling time point, ZT18 (night), most neurons are electrically silent, or fire at a low frequency (~ 2.5 Hz). By increasing the extracellular K^+^, the membrane potential was depolarized, thereby increasing neuronal firing. **c** Exposure to high K^+^ at midnight severely upregulated all metabolites of the TCA cycle. The difference between midday and midnight was smaller, with one of the five measurable metabolites upregulated

Metabolites were measured in SCN and PVN tissue at midday (ZT6) and midnight (ZT18). In the SCN there is a strong circadian rhythm in neuronal activity, which peaks at midday and is lowest at midnight (Inouye and Kawamura 1979; Sato and Kawamura 1984). In vitro MUA recordings in the PVN have shown that its neuronal activity depends on AVP released from the SCN, but that the rhythm of electrical activity is in phase with that of the SCN (Tousson and Meissl 2004). To assess the impact of neuronal activity on metabolites, firing frequency was modulated in the SCN and PVN to simulate either a midday or midnight situation (Fig. 4). For this purpose, TTX was applied to silence the neurons of the SCN and PVN during the day, and elevated extracellular K^+^ was applied to depolarize the neurons and increase firing frequency during the night. We found that the exposure to a higher K^+^ concentration significantly upregulated all measured intermediates of the TCA-cycle in both the SCN and PVN. This suggests a higher energy demand of the neurons, as is previously described (Shetty et al. 2012). Consequently, we expected glycolysis to be upregulated in these samples, however, no significant differences in glycolysis intermediates was observed (Fig. S2). This indicates that different pathways from either fatty acids (fatty acid oxidation) or amino acids are used for energy metabolism under these conditions.

Only one of the measurable TCA cycle intermediates we found was significantly different between midday and midnight, and none were significantly different between midday and TTX treatment. This is surprising since cells are also more electrically active during the day than during the night or when silenced by with TTX (Shibata et al. 1984, 1982). However, the action potential frequency in SCN neurons is overall relatively low compared to other neurons, with a maximum of around 5 Hz during the day, and around 2.5 Hz during the night (Schaap et al. 1999). We do see a trend towards upregulation of the metabolites in the control-day group, compared to TTX treated and control-night samples (Fig. 2b). However, it is clear that high K^+^ upregulates most of the metabolites we measured more severely than is the case for control-day vs TTX-day or control-night. An explanation could be that there is a greater increase in electrical activity as a consequence of the manipulation with high K^+^, compared to normal electrical activity at midday. This is perceivable, since high K^+^ pulses have been used to mimic light pulses (Eskin 1972; Mirmiran et al. 1995; Schwartz 1991), and light pulses can elicit a response in firing frequency up to 25 Hz in SCN neurons (Irwin and Allen 2007; Meijer et al. 1998). An additional explanation could be that there is a homeostatic control of energy metabolites to anticipate the change in energy demand from night to day. In that case silencing the neurons with TTX might have a minimal effect on metabolite levels. Furthermore, individual neurons are naturally also inactive for a large proportion of the day; they are generally only active for about 5 h during daytime, and silent for the remaining 19 h (Schaap et al. 2003; VanderLeest et al. 2007).

The relatively strong response to the manipulation of electrical activity at midnight may be linked to the circadian fluctuation in the sensitivity to light pulses (Meijer et al. 1998). Light signals that reach the SCN from the eye through the RHT elicit electrical responses in SCN neurons. Experiments both in vivo-with light exposure—as well as in vitro-by electrically stimulating the RHT—have shown that this only results in a phase shift in behaviour or in the electrical activity rhythm when received during the subjective night, not when the same signal is received during the subjective day (Shirakawa and Moore 1994). It could be that the strong response in metabolite levels to increased electrical activity at midnight is a means for the cells to support intracellular signalling pathways that generate light-induced phase shifts of the molecular clock.

The absence of detectible differences between metabolite levels at midday and midnight can have several reasons. First, if the peak and trough of the waveform is 6 h shifted from our sampling time points, there will be no differences detected. If the peak and trough are anywhere between 0 and 6 h from the sampling time points, this will result in smaller differences between the two measurements. Furthermore, the SCN and PVN are heterogeneous population of cells—including different types of neurons, as well as astrocytes—with diverse phases in their individual circadian rhythms (Abrahamson and Moore 2001; Brancaccio et al. 2017; Enoki et al. 2017; Tasker and Dudek 1991). Therefore, metabolites that are rhythmic in single cells, can be arrhythmic at the tissue level. Lastly, the one-hour incubation in ACSF may influence metabolite level in some unanticipated way. However, this is unlikely, since this incubation time does not affect rhythms in other physiological parameters of (SCN) neurons, like membrane potential, firing rate, intracellular Ca^2+^ levels or synaptic activity (Nakamura et al. 2012).

In this study, we looked at static basic metabolism. To gain more information on the involvement of energy balance, metabolic flexibility and substrate specificity of the SCN and PVN tissue, it could be interesting to follow metabolic fluxes by using isotopically stable labelled metabolites through the neuronal networks of the SCN and PVN under similar conditions as was described in this study. Using this technique, it would be possible to determine the real flux for the TCA cycle and which energy metabolites, glucose, fatty acids or amino acids, are involved for the energy utilization in neurons under high K^+^ stimulation.

We show here that it is feasible to measure metabolites in small samples of brain tissue with a broadly applicable method, making it usable to tackle diverse biological questions. Using this method, we show that time of day, and manipulation of electrical activity affects metabolite levels in SCN and PVN tissue. Increasing electrical activity at midnight strongly upregulates metabolites of the TCA cycle. Our metabolomic study in the central circadian clock offers a unique view into the cellular biochemistry. Metabolites are not only a consequence of physiological states, they can actively influence cellular signalling pathways, including those involved in clock functioning. By combining this analytical tool with endogenous and exogenous modulation of neuronal activity, we were able to show the differential effect of these two sources of excitability on metabolites involved in cellular energy balance and signalling in the SCN.

## Electronic supplementary material

Below is the link to the electronic supplementary material.

Supplementary material 1 (PDF 537 KB)

## Data Availability

The datasets generated during and/or analysed during the current study are available from the corresponding author on reasonable request.

## References

[CR1] Abrahamson EE, Moore RY (2001). Suprachiasmatic nucleus in the mouse: Retinal innervation, intrinsic organization and efferent projections. Brain Research.

[CR2] Allen Charles N., Nitabach Michael N., Colwell Christopher S. (2017). Membrane Currents, Gene Expression, and Circadian Clocks. Cold Spring Harbor Perspectives in Biology.

[CR3] Brancaccio M, Patton AP, Chesham JE, Maywood ES, Hastings MH (2017). Astrocytes control circadian timekeeping in the suprachiasmatic nucleus via glutamatergic signaling. Neuron.

[CR4] Chong I-G, Jun C-H (2005). Performance of some variable selection methods when multicollinearity is present. Chemometrics and Intelligent Laboratory Systems.

[CR5] Colwell CS (2011). Linking neural activity and molecular oscillations in the SCN. Nature Reviews.

[CR6] Coomans CP, van den Berg SAA, Lucassen EA, Houben T, Pronk ACM, van der Spek RD, Kalsbeek A, Biermasz NR, van WillemsDijk K, Romijn JA, Meijer JH (2013). The suprachiasmatic nucleus controls circadian energy metabolism and hepatic insulin sensitivity. Diabetes.

[CR7] Davies SK, Ang JE, Revell VL, Holmes B, Mann A, Robertson FP, Cui N, Middleton B, Ackermann K, Kayser M, Thumser AE, Raynaud FI, Skene DJ (2014). Effect of sleep deprivation on the human metabolome. Proceedings of the National Academy of Sciences.

[CR8] Eckel-Mahan KL, Patel VR, Mohney RP, Vignola KS, Baldi P, Sassone-Corsi P (2012). Coordination of the transcriptome and metabolome by the circadian clock. Proceedings of the National Academy of Sciences.

[CR9] Enoki R, Oda Y, Mieda M, Ono D, Honma S, Honma KI (2017). Synchronous circadian voltage rhythms with asynchronous calcium rhythms in the suprachiasmatic nucleus. Proceedings of the National Academy of Sciences of the United States of America.

[CR10] Eskin A (1972). Phase shifting a circadian rhythm in the eye of Aplysia by high potassium pulses. Journal of Comparative Physiology.

[CR11] Gillette MU, Reppert SM (1987). The hypothalamic suprachiasmatic nuclei: Circadian patterns of vasopressin secretion and neuronal activity in vitro. Brain Research Bulletin.

[CR12] Ibuka N, Kawamura H (1975). Loss of circadian rhythm in sleep-wakefulness cycle in the rat by suprachiasmatic nucleus lesions. Brain Research.

[CR13] Ikeda M, Sugiyama T, Wallace CS, Gompf HS, Yoshioka T, Miyawaki A, Allen CN (2003). Circadian dynamics of cytosolic and nuclear Ca^2+^ in single suprachiasmatic nucleus neurons. Neuron.

[CR14] Inouye ST, Kawamura H (1979). Persistence of circadian rhythmicity in a mammalian hypothalamic “island” containing the suprachiasmatic nucleus. Proceedings of the National Academy of Sciences.

[CR15] Irwin RP, Allen CN (2007). Calcium response to retinohypothalamic tract synaptic transmission in suprachiasmatic nucleus neurons. Journal of Neuroscience.

[CR16] Ju Y-E S, Lucey BP, Holtzman DM (2013). Sleep and Alzheimer disease pathology: A bidirectional relationship. Nature Reviews Neurology.

[CR17] Kalsbeek A, La Fleur S, Van Heijningen C, Buijs RM (2004). Suprachiasmatic GABAergic inputs to the paraventricular nucleus control plasma glucose concentrations in the rat via sympathetic innervation of the liver. Journal of Neuroscience.

[CR18] Kalsbeek A, Scheer FA, Perreau-Lenz S, La Fleur SE, Yi CX, Fliers E, Buijs RM (2011). Circadian disruption and SCN control of energy metabolism. FEBS Letters.

[CR19] Kondratova AA, Kondratov RV (2012). The circadian clock and pathology of the ageing brain. Nature Reviews.

[CR20] Kubota A, Inouye S-I T, Kawamura H (1981). Reversal of multiunit activity within and outside the suprachiasmatic nucleus in the rat. Neuroscience Letters.

[CR21] Lapainis T, Rubakhin SS, Sweedler JV (2009). Capillary electrophoresis with electrospray ionization mass spectrometric detection for single-cell metabolomics. Analytical Chemistry.

[CR22] Lee JE, Atkins N, Hatcher NG, Zamdborg L, Gillette MU, Sweedler JV, Kelleher NL (2010). Endogenous peptide discovery of the rat circadian clock: A focused study of the suprachiasmatic nucleus by ultrahigh performance tandem mass spectrometry. Molecular and Cellular Proteomics.

[CR23] Meijer JH, Watanabe K, Schaap J, Albus H, Detari L (1998). Light responsiveness of the suprachiasmatic nucleus: Long-term multiunit and single-unit recordings in freely moving rats. Journal of Neuroscience.

[CR24] Michel S, Marek R, Vanderleest HT, Vansteensel MJ, Schwartz WJ, Colwell CS, Meijer JH (2013). Mechanism of bilateral communication in the suprachiasmatic nucleus. European Journal of Neuroscience.

[CR25] Minami Y, Kasukawa T, Kakazu Y, Iigo M, Sugimoto M, Ikeda S, Yasui A, van der Horst GTJ, Soga T, Ueda HR (2009). Measurement of internal body time by blood metabolomics. Proceedings of the National Academy of Sciences.

[CR26] Mirmiran M, Koster-Van Hoffen GC, Bos NPA (1995). Circadian rhythm generation in the cultured suprachiasmatic nucleus. Brain Research Bulletin.

[CR27] Moore RY (2007). Suprachiasmatic nucleus in sleep-wake regulation. Sleep Medicine.

[CR28] Moore RY, Eichler VB (1972). Loss of a circadian adrenal corticosterone rhythm following suprachiasmatic lesions in the rat. Brain Research.

[CR29] Musiek ES, Xiong DD, Holtzman DM (2015). Sleep, circadian rhythms, and the pathogenesis of Alzheimer disease. Experimental and Molecular Medicine.

[CR30] Nagai K, Nishio T, Nakagawa H, Nakamura S, Fukuda Y (1978). Effect of bilateral lesions of the suprachiasmatic nuclei on the circadian rhythm of food-intake. Brain Research.

[CR31] Nakamura TJ, Michel S, Block GD, Colwell CS, Ballanyi K (2012). Neural circuits underlying circadian oscillations in mammals: Clocks in a dish. Isolated central nervous system circuits.

[CR32] Nemes P, Knolhoff AM, Rubakhin SS, Sweedler JV (2011). Metabolic differentiation of neuronal phenotypes by single-cell capillary electrophoresis–electrospray ionization-mass spectrometry. Analytical Chemistry.

[CR33] Nishino H, Kiyomi K, Brooks CM (1976). The role of suprachiasmatic nuclei of the hypothalamus in the production of circadian rhythm. Brain Research.

[CR34] O’Neill JS, Maywood ES, Chesham JE, Takahashi JS, Hastings MH (2008). cAMP-dependent signaling as a core component of the mammalian circadian pacemaker. Science.

[CR36] Qi M, Philip MC, Yang N, Sweedler JV (2018). Single cell neurometabolomics. ACS Chemical Neuroscience.

[CR37] Rey G, Valekunja UK, Feeney KA, Wulund L, Milev NB, Stangherlin A, Ansel-Bollepalli L, Velagapudi V, O’Neill JS, Reddy AB (2016). The pentose phosphate pathway regulates the circadian clock. Cell Metab.

[CR35] Rohart F, Gautier B, Singh A, Lê Cao K-A (2017). mixOmics: An R package for ‘omics feature selection and multiple data integration. PLoS Computational Biology.

[CR38] Rudic RD, McNamara P, Curtis AM, Boston RC, Panda S, Hogenesch JB, Fitzgerald GA (2004). BMAL1 and CLOCK, two essential components of the circadian clock, are involved in glucose homeostasis. PLoS Biology.

[CR39] Sangoram AM, Saez L, Antoch MP, Gekakis N, Staknis D, Whiteley A, Fruechte EM, Vitaterna MH, Shimomura K, King DP, Young MW, Weitz CJ, Takahashi JS (1998). Mammalian circadian autoregulatory loop: A timeless ortholog and mPer1 interact and negatively regulate CLOCK-BMAL1-induced transcription. Neuron.

[CR40] Santoso P, Nakata M, Ueta Y, Yada T (2017). Suprachiasmatic vasopressin to paraventricular oxytocin neurocircuit in the hypothalamus relays light reception to inhibition of feeding behavior. American Journal of Physiology-Endocrinology and Metabolism.

[CR41] Sato T, Kawamura H (1984). Circadian rhythms in multiple unit activity inside and outside the suprachiasmatic nucleus in the diurnal chipmunk (Eutamias sibiricus). Neuroscience Research.

[CR42] Schaap J, Albus H, vanderLeest HT, Eilers PHC, Détári L, Meijer JH (2003). Heterogeneity of rhythmic suprachiasmatic nucleus neurons: Implications for circadian waveform and photoperiodic encoding. Proceedings of the National Academy of Sciences.

[CR43] Schaap J, Bos NP, de Jeu MT, Geurtsen AM, Meijer JH, Pennartz CM (1999). Neurons of the rat suprachiasmatic nucleus show a circadian rhythm in membrane properties that is lost during prolonged whole-cell recording. Brain Research.

[CR44] Schwartz WJ (1991). Further evaluation of the tetrodotoxin-resistant circadian pacemaker in the suprachiasmatic nuclei. Journal of Biological Rhythms.

[CR45] Shetty, P. K., Galeffi, F., & Turner, D. A. (2012) Cellular links between neuronal activity and energy homeostasis. *Frontiers in Pharmacology* 3. 10.3389/fphar.2012.00043 PMC3308331 22470340

[CR46] Shibata S, Oomura Y, Hattori K, Kita H (1984). Responses of suprachiasmatic nucleus neurons to optic nerve stimulation in rat hypothalamic slice preparation. Brain Research.

[CR47] Shibata S, Oomura Y, Kita H, Hattori K (1982). Circadian rhythmic changes of neuronal activity in the suprachiasmatic nucleus of the rat hypothalamic slice. Brain Research.

[CR48] Shirakawa T, Moore RY (1994). Glutamate shifts the phase of the circadian neuronal firing rhythm in the rat suprachiasmatic nucleus in vitro. Neuroscience Letters.

[CR49] Stephan FK, Zucker I (1972). Circadian rhythms in drinking behavior and locomotor activity of rats are eliminated by hypothalamic lesions. Proceedings of the National Academy of Sciences.

[CR50] Tasker JG, Dudek FE (1991). Electrophysiological properties of neurones in the region of the paraventricular nucleus in slices of rat hypothalamus. The Journal of Physiology.

[CR51] Tousson E, Meissl H (2004). Suprachiasmatic nuclei grafts restore the circadian rhythm in the paraventricular nucleus of the hypothalamus. Journal of Neuroscience.

[CR52] Turek FW, Joshu C, Kohsaka A, Lin E, Ivanova G, McDearmon E, Laposky A, Losee-Olson S, Easton A, Jensen DR, Eckel RH, Takahashi JS, Bass J (2005). Obesity and metabolic syndrome in circadian Clock mutant mice. Science.

[CR53] VanderLeest HT, Houben T, Michel S, Deboer T, Albus H, Vansteensel MJ, Block GD, Meijer JH (2007). Seasonal encoding by the circadian pacemaker of the SCN. Current Biology.

[CR54] Wang TA, Yu YV, Govindaiah G, Ye X, Artinian L, Coleman TP, Sweedler JV, Cox CL, Gillette MU (2012). Circadian rhythm of redox state regulates excitability in suprachiasmatic nucleus neurons. Science.

[CR55] Wulff K, Gatti S, Wettstein JG, Foster RG (2010). Sleep and circadian rhythm disruption in psychiatric and neurodegenerative disease. Nature Reviews.

[CR56] Zhu J, Djukovic D, Deng L, Gu H, Himmati F, Chiorean EG, Raftery D (2014). Colorectal cancer detection using targeted serum metabolic profiling. Journal of Proteome Research.

