# Supplementary material for: The influence of neuronal electrical activity on the mammalian central clock metabolome
Source: Metabolomics. 2018 Sep 17;14(10):122. doi: 10.1007/s11306-018-1423-z (PMC6153692; doi:10.1007/s11306-018-1423-z)
Supplement: Supplementary file 1 — Supplementary material 1 (PDF 537 KB) [file 11306_2018_1423_MOESM1_ESM.pdf]

Supplemental table 1: LOD, LOQ and used IS for metabolite analysis

METABOLITES	MSI level	InChI	RT (min)	LOD (µM)	LLOQ (µM)	IS correction
**2- or 3-phosphoglyceric acid**	2	GXIURPTVHJPJLF-UHFFFAOYSA-N<br>OSJPPGNTCRNQQC-REOHCLBHSA-N	14.75	ND	ND	ISTD - UMP-15N2
**4-Aminobutyric acid (GABA)**	2	BTCSSZJGUNDROE-UHFFFAOYSA-N	14.94	ND	ND	ISTD - Succinic acid-D4
**ADP**	1	XTWYTFMLZFPYCI-KQYNXXCUSA-N	12.45	0.02	0.07	ISTD - Glutamine-13C5
**AMP**	1	UDMBCSSLTHHNCD-KQYNXXCUSA-N	12.02	0.01	0.03	ISTD - UMP-15N2
**ATP**	1	ZKHQWZAMYRWXGA-KQYNXXCUSA-N	12.92	0.02	0.05	ISTD - Valine-13C5
**cis-Aconitic acid**	1	GTZCVFVGUGFEME-IWQZZHSRSA-N	11.79	0.04	0.13	ISTD - Glutamine-13C5
**Citric acid**	2	KRKNYBCHXYNGOX-UHFFFAOYSA-N	15.28	ND	ND	ISTD - UMP-15N2
**CMP**	1	IERHLVCPSMICTF-XVFCMESISA-L	12.62	0.03	0.09	ISTD - Glutamine-13C5
**CTP**	1	PCDQPRRSZKQHHS-XVFCMESISA-N	13.35	0.03	0.09	ISTD - UMP-15N2
**Fructose-1,6-bisphosphate**	2	RNBGYGVWRKECFJ-ZXXMMSQZSA-N	15.43	ND	ND	ISTD - UMP-15N2
**Fumaric acid**	1	VZCYOOQTPOCHFL-OWOJBTEDSA-N	11.86	0.27	0.88	ISTD - UMP-15N2
**GDP**	1	RQFCJASXJCIDSX-UUOKFMHZSA-N	13.05	0.00	0.01	ISTD - UMP-15N2
**Gluconic acid 6-phosphate**	1	ZKUSPPOKDDRMIU-JJYYJPOSSA-N	12.99	0.02	0.07	ISTD - UMP-15N2
**Glucose**	1	GZCGUPFRVQAUEE-SLPGGIOYSA-N	12.81	ND	ND	ISTD - Glutamine-13C5
**Glucose-6-phosphate**	1	NBSCHQHZLSJFNQ-GASJEMHNSA-N	15.11	ND	ND	ISTD - UMP-15N2
**Glutathione**	1	RWSXRVCMGQZWBV-WDSKDSINSA-N	11.97	0.01	0.04	ISTD - Glutamine-13C5
**Glyceraldehyde-3-phosphate**	2	LXJXRIRHZLFYRP-UHFFFAOYSA-N	14.50	ND	ND	ISTD - UMP-15N2
**Glycerol 3-phosphate**	1	AWUCVROLDVIAJX-UHFFFAOYSA-N	12.52	0.02	0.06	ISTD - Glutamine-13C5
**GMP**	1	RQFCJASXJCIDSX-UUOKFMHZSA-N	12.88	0.03	0.08	ISTD - Asparagine-13C4 15N2
**GTP**	1	XKMLYUALXHKNFT-UUOKFMHZSA-N	13.48	0.12	0.39	ISTD - UMP-15N2
**Hypoxanthine**	1	FDGQSTZJBFJUBT-UHFFFAOYSA-N	5.07	0.05	0.18	ISTD - UMP-15N2
**IMP**	1	GRSZFWQUAKGDAV-KQYNXXCUSA-N	12.40	0.01	0.04	ISTD - UMP-15N2
**Lactic acid**	1	LPEKGGXMPWTOCB-GSVOUGTGSA-N<br>LPEKGGXMPWTOCB-VKHMYHEASA-N	8.44	1.09	3.26	ISTD - UMP-15N2
**Malic acid**	1	BJEPYKJPYRNKOW-UHFFFAOYSA-N	12.05	0.03	0.10	ISTD - Glutamine-13C5

**NAD+ (oxidized)**	1	BAWFJGJZGIEFAR-NNYOXOHSSA-N	12.30	0.00	0.01	ISTD - Valine-13C5
**NADH (reduced)**	1	BOPGDPNILDQYTO-NNYOXOHSSA-N	11.72	0.00	0.00	ISTD - UMP-15N2
**NADP+ (oxidized)**	1	XJLXINKUBYWONI-NNYOXOHSSA-O	13.14	0.01	0.03	ISTD - Valine-13C5
**Oxiglutathione**	1	YPZRWBKMTBYPTK-BJDJZHNGSA-N	13.36	0.04	0.13	ISTD - UMP-15N2
**PEP**	1	DTBNBXWJWCWCIK-UHFFFAOYSA-N	12.84	0.00	0.01	ISTD - UMP-15N2
**Sedoheptulose 7-phosphate**	1	JDTUMPKOJBQPKX-GBNDHIKLSA-N	13.00	0.01	0.03	ISTD - UMP-15N2
**Succinic acid**	1	KDYFGRWQOYBRFD-UHFFFAOYSA-N	11.77	0.70	2.32	ISTD - Glutamine-13C5
**UDP**	1	XCCTYIAWTASOJW-XVFCMESISA-N	12.53	0.01	0.04	ISTD - Glutamine-13C5
**UMP**	1	DJJCXFVJDGTHFX-XVFCMESISA-N	12.18	0.10	0.33	ISTD - UMP-15N2
**Uridine**	1	DRTQHJPVMGBUCF-XVFCMESISA-N	5.19	0.00	0.01	ISTD - Asparagine-13C4 15N2
**UTP**	1	PGAVKCOVUIYSFO-XVFCMESISA-N	13.01	0.01	0.04	ISTD - Valine-13C5

Supplemental fig. 1

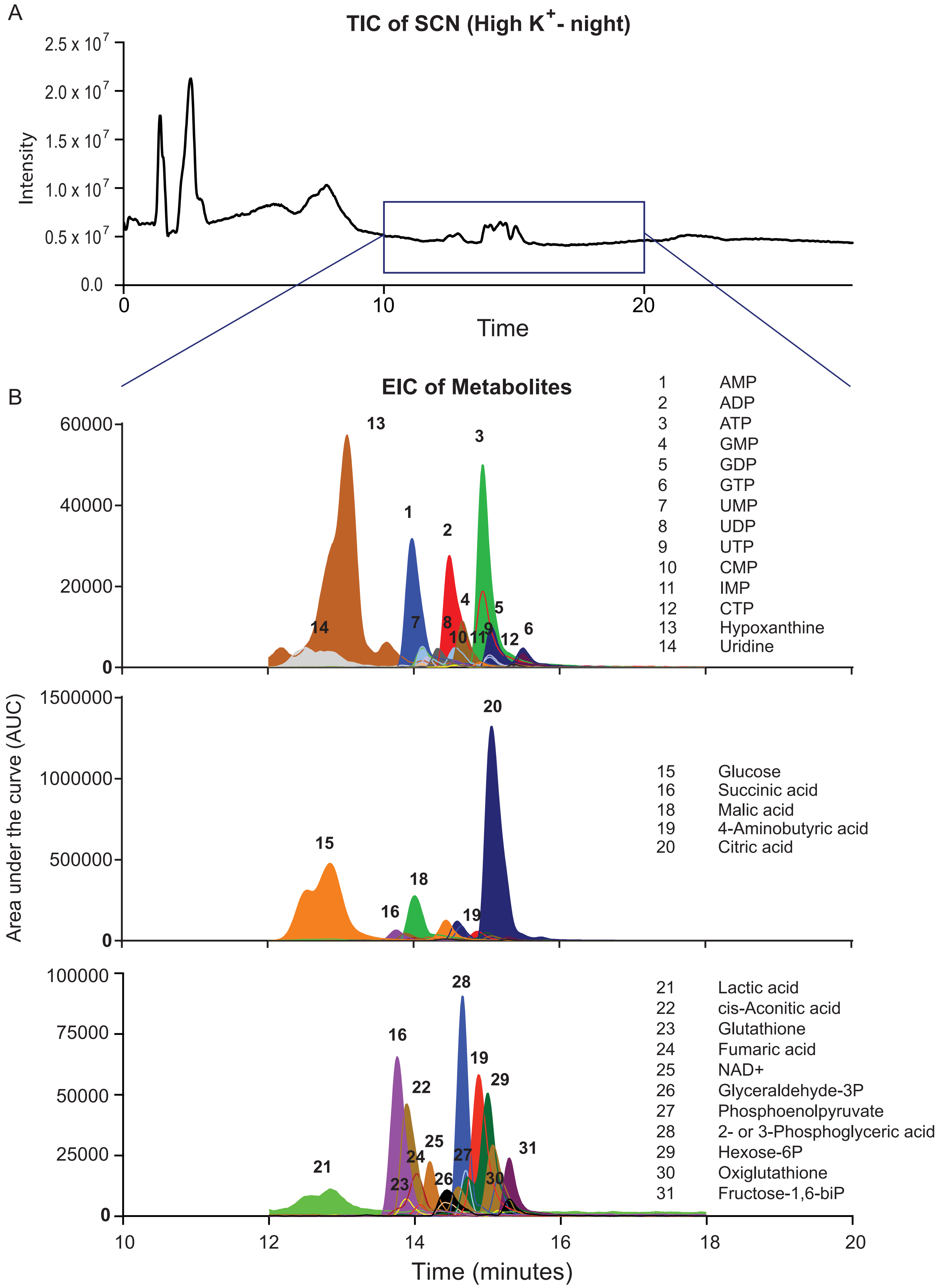

Supplemental fig. 1 ZIC-cHILIC-MS analysis of the SCN metabolome. a Total ion chromatogram (TIC) of a SCN night (with high K) sample. Most of the metabolites elute from the ZIC-cHILIC column between 10 and 20 minutes. b Extracted ion chromatograms (EIC) of most of the identified metabolite classes between 10 and 20 minutes in a SCN night (with high K) sample.

Supplemental fig. 2

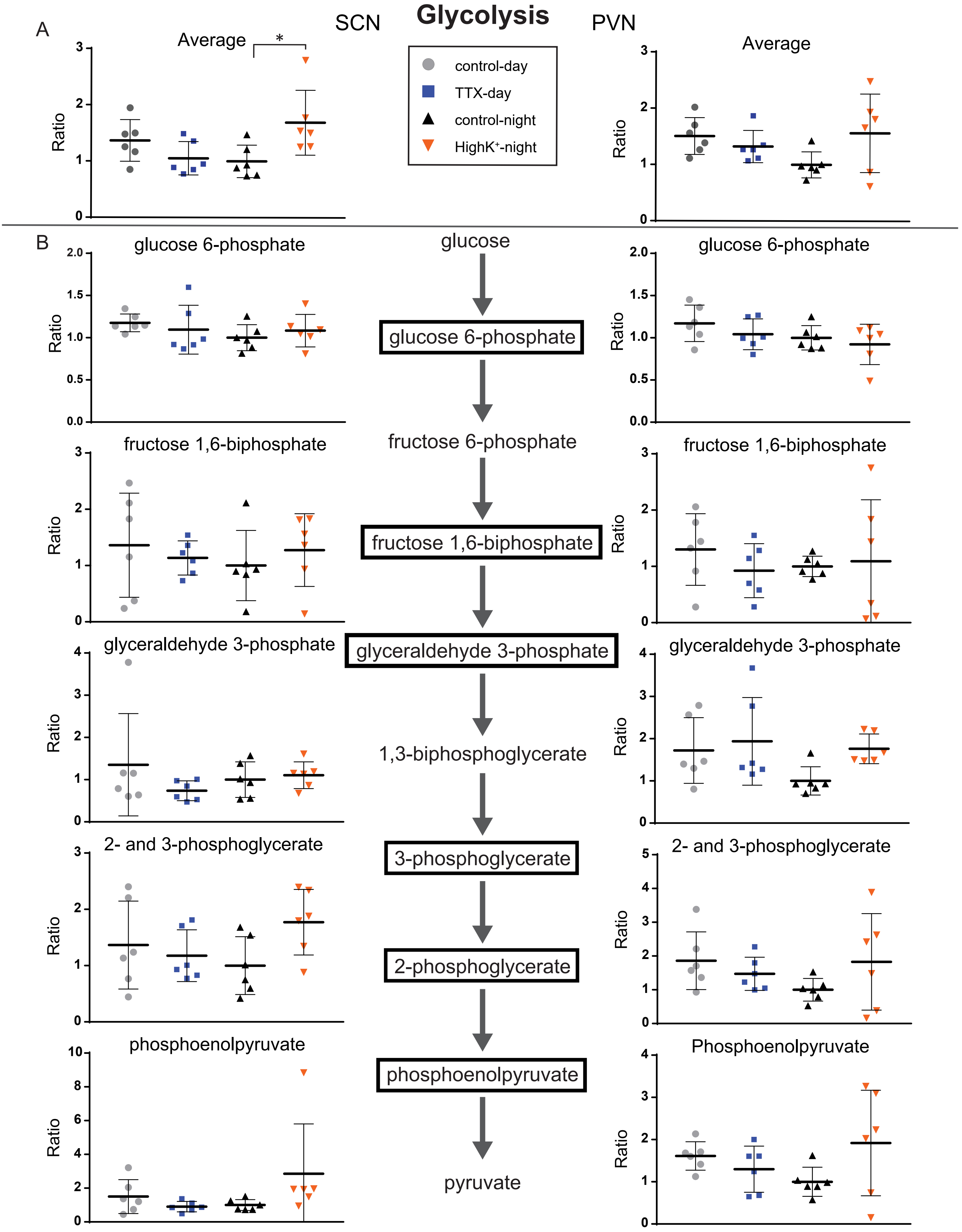

Supplemental fig. 2 Metabolites of the glycolysis are not affected by time of day or manipulation of electrical activity in both the SCN and PVN. a Only averaged levels showed a small, but significant difference between the control-night and high K+-night condition in the SCN. b From the main metabolites of the glycolysis, six were reliably measurable with the ZIC-cHILIC-MS method (boxed). We detected no differences in individual metabolite levels between the 4 groups in both the SCN and PVN. * = $P < 0.05$.

Supplemental fig. 3a

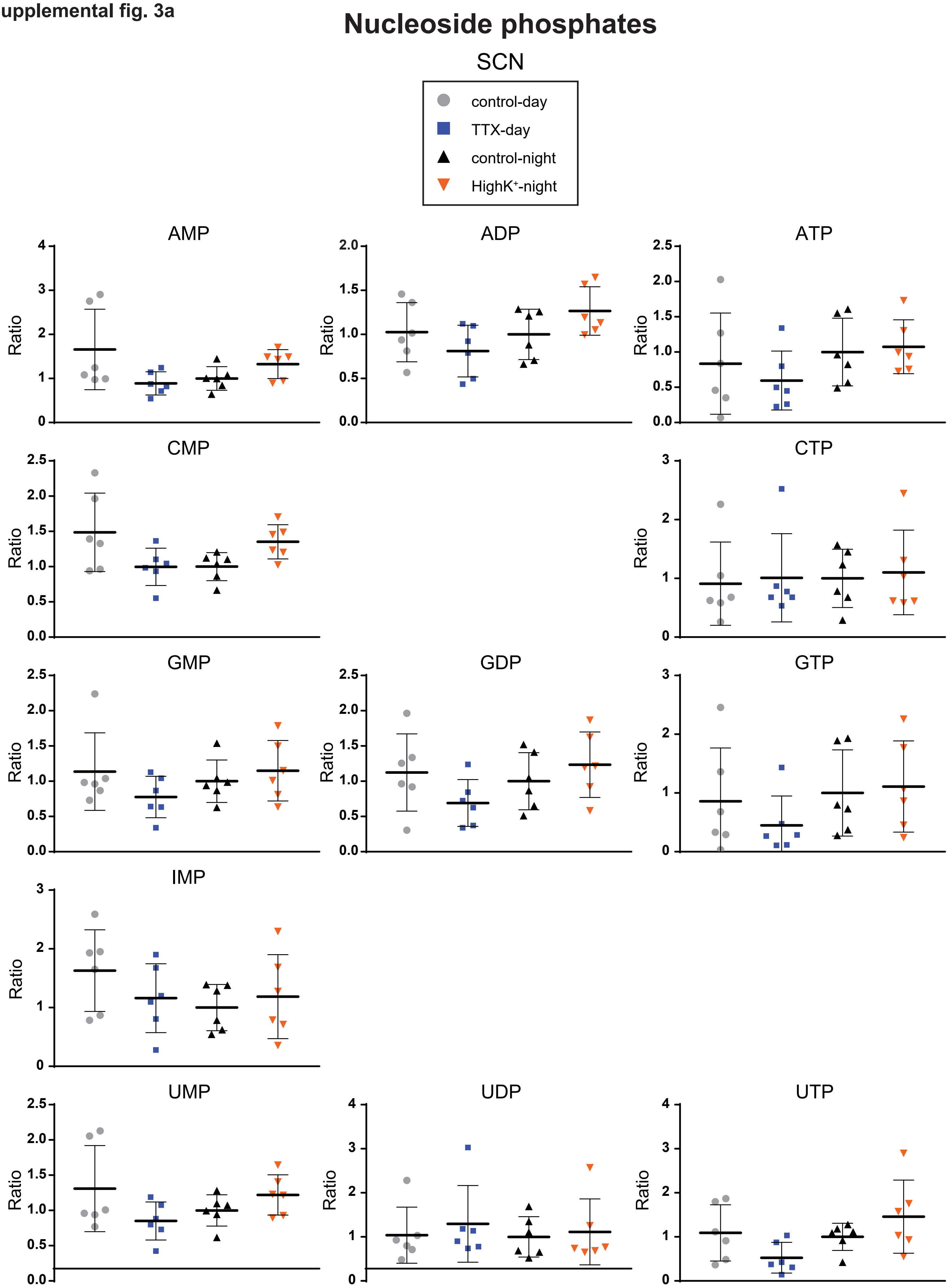

Supplemental fig. 3a Nucleoside phosphate metabolites in the SCN. Overview of all measured nucleoside phosphate metabolites in SCN tissue. For these metabolites, none of the groups that were compared showed significant effects (control day – control night, control day – TTX day, control night – High K+ night). Data was analysed using one-way ANOVA corrected for multiple comparisons with Holm-Sidak.

Supplemental fig. 3b

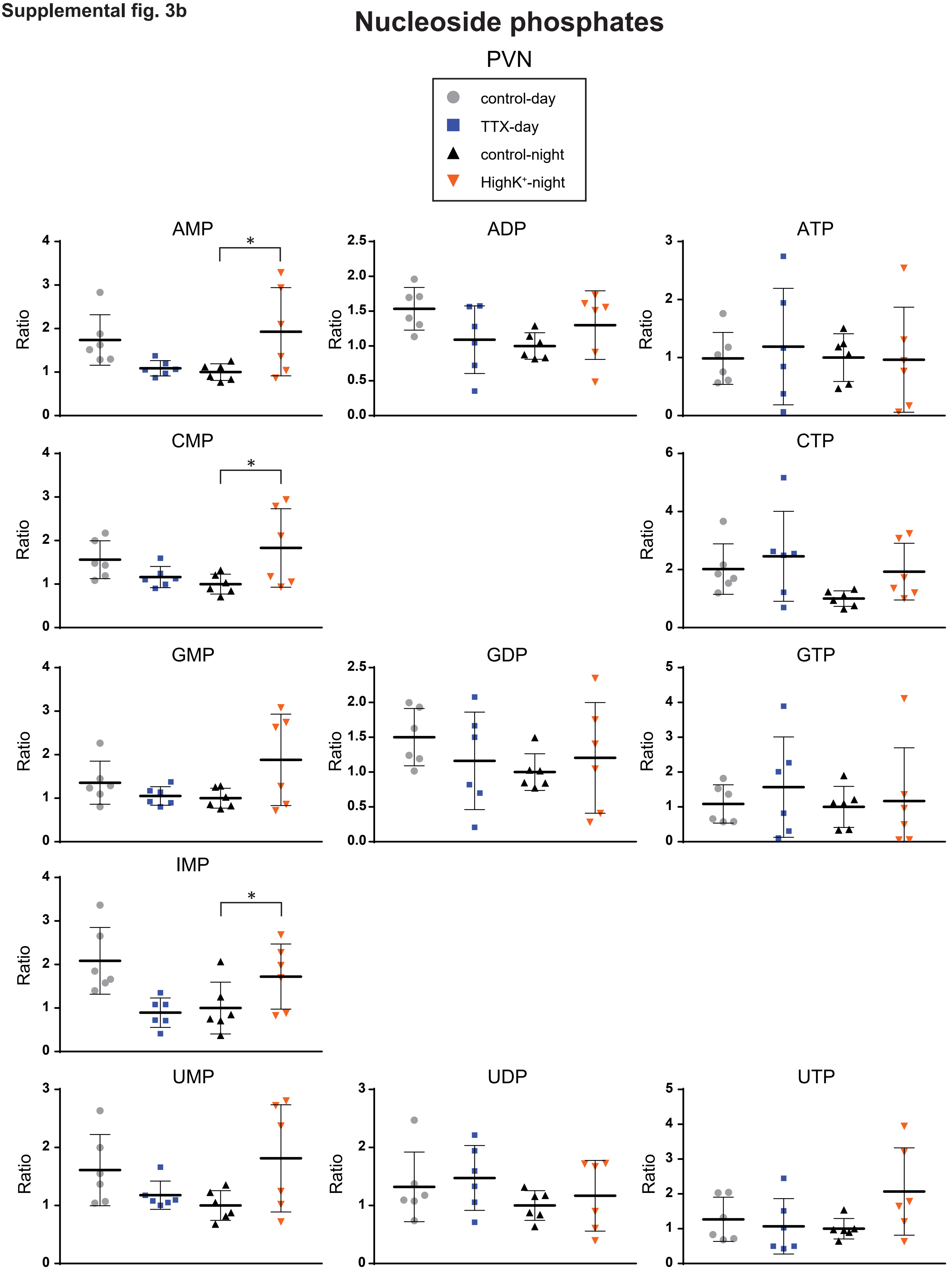

Supplemental fig. 3b Nucleoside phosphate metabolites in the PVN. Overview of all measured nucleoside phosphate metabolites in PVN tissue. Three monophosphates, AMP, CMP and IMP showed a significant higher level in the high K+ night group, compared to control night. None of the other metabolites showed significant effects for the groups that were compared (control day – control night, control day – TTX day, control night – high K+ night). Data was analysed using one-way ANOVA, corrected for multiple comparisons with Holm-Sidak. * = $P < 0.05$.

Supplemental fig. 4

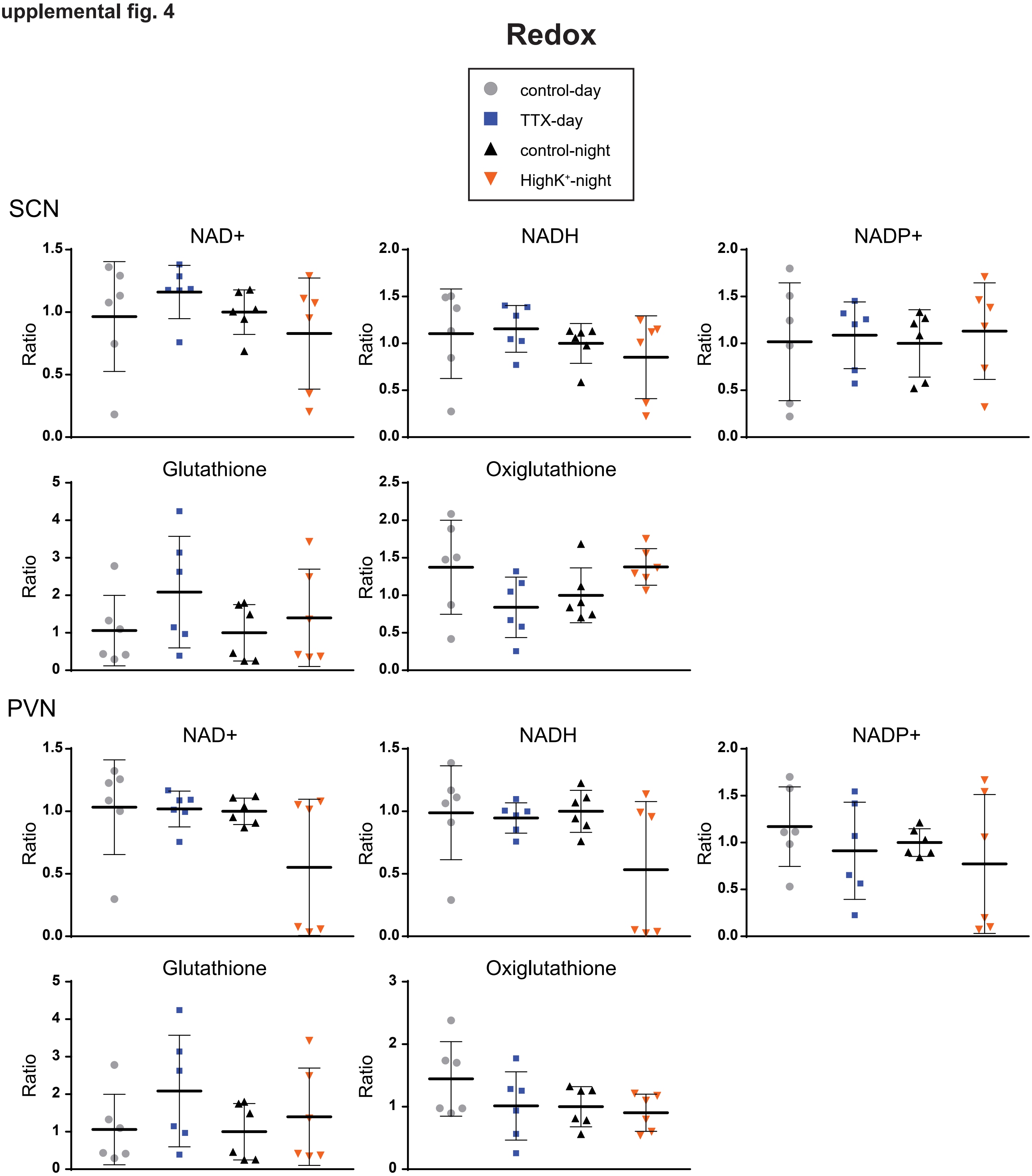

Supplemental fig. 4 Redox associated metabolites in the SCN and PVN. Overview of five redox associated metabolites measured in the SCN and PVN. For these metabolites, none of the groups that were compared showed significant effects (control day – control night, control day – TTX day, control night – High K+ night). Data was analysed using one-way ANOVA, corrected for multiple comparisons with Holm-Sidak.

Supplemental fig. 5

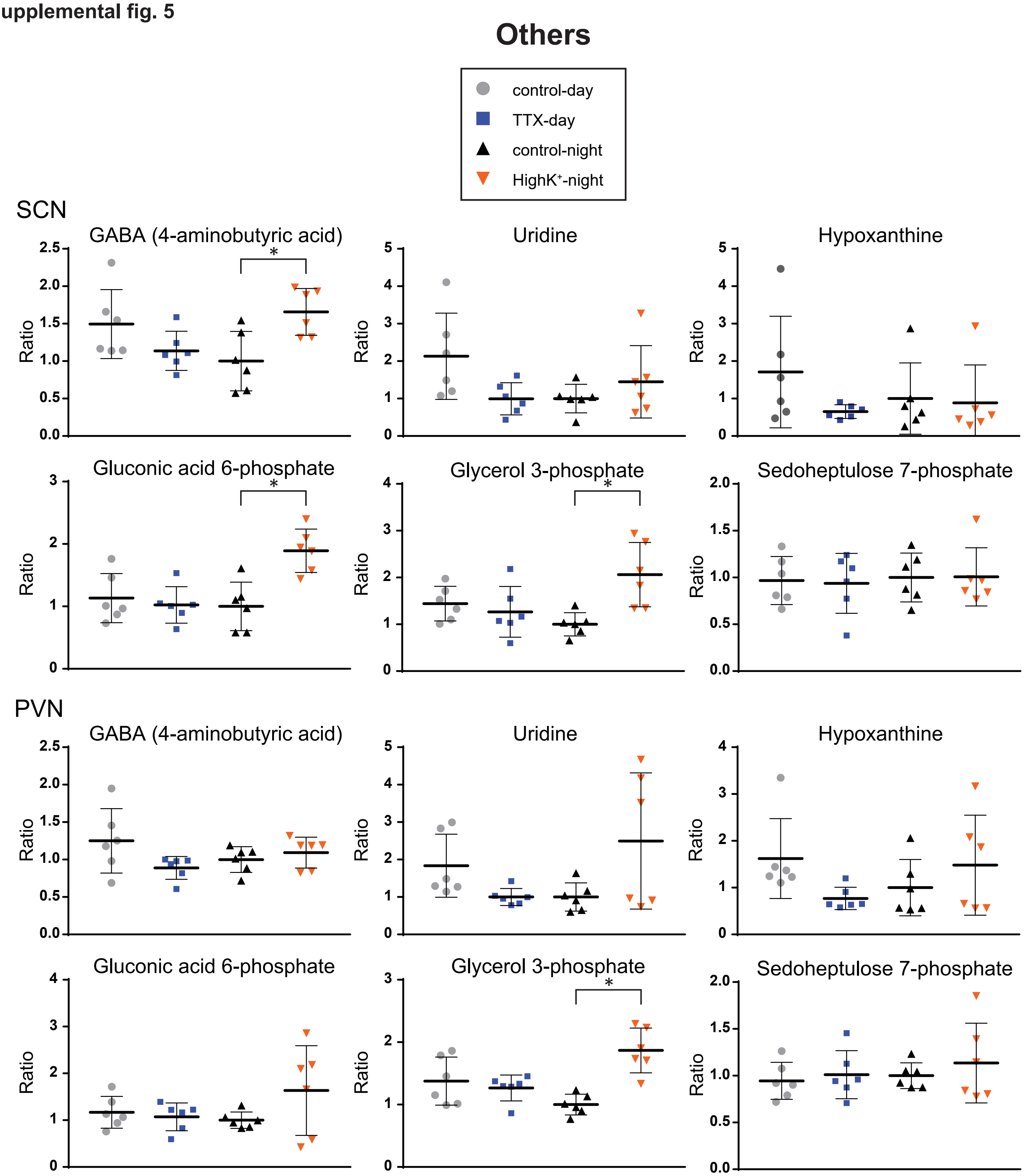

Supplemental fig. 5 Other metabolites measured in the SCN and PVN. Overview of metabolites not belonging to the other groups above. For the SCN, GABA (4-aminobutyric acid), gluconic acid 6-phosphate and glycerol 3-phosphate showed a significant upregulation in the high K+ night group, compared to control night. For the PVN, glycerol 3-phosphate showed a significant upregulation in the high K+ night group, compared to control night. None of the other metabolites showed significant effects for the groups that were compared (control day – control night, control day – TTX day, control night – high K+ night). Data was analysed using one-way ANOVA, corrected for multiple comparisons with Holm-Sidak. * = $P < 0.05$.
